# Supplementary material for: SARS CoV-2 (Delta Variant) Infection Kinetics and Immunopathogenesis in Domestic Cats
Source: Viruses. 2022 Jun 1;14(6):1207. doi: 10.3390/v14061207 (PMC9230585; doi:10.3390/v14061207)
Supplement: Supplementary file 1 [file viruses-14-01207-s001.zip › viruses-1737675-supplementary-new/Tamil Selvan et al. Supplementary Figures.pdf]

Supplementary Figures

Figure S1. Timeline of *in vivo* studies

Figure S2. Clustering dendrogram of RNA Seq samples

Figure S3. Clinical signs of SARS-CoV-2 cats by inoculation route

Figure S4. Nasal swab cultures

Figure S5. Histologic and immunofluorescent scoring of lung tissues in SARS-CoV-2 infected animals.

Figure S6. SARS-CoV-2 viral loads in necropsy tissues by inoculation route

Figure S7. Feline ACE2 expression in necropsy tissues by inoculation route

Figure S8A-C. Differentially expressed genes in control and CoV-2+ lung samples

Figure S9. Gene modules detection by weighted correlation network analysis (WGCNA) analysis

Figure S10A-D. KEGG pathway and gene ontology term enrichment

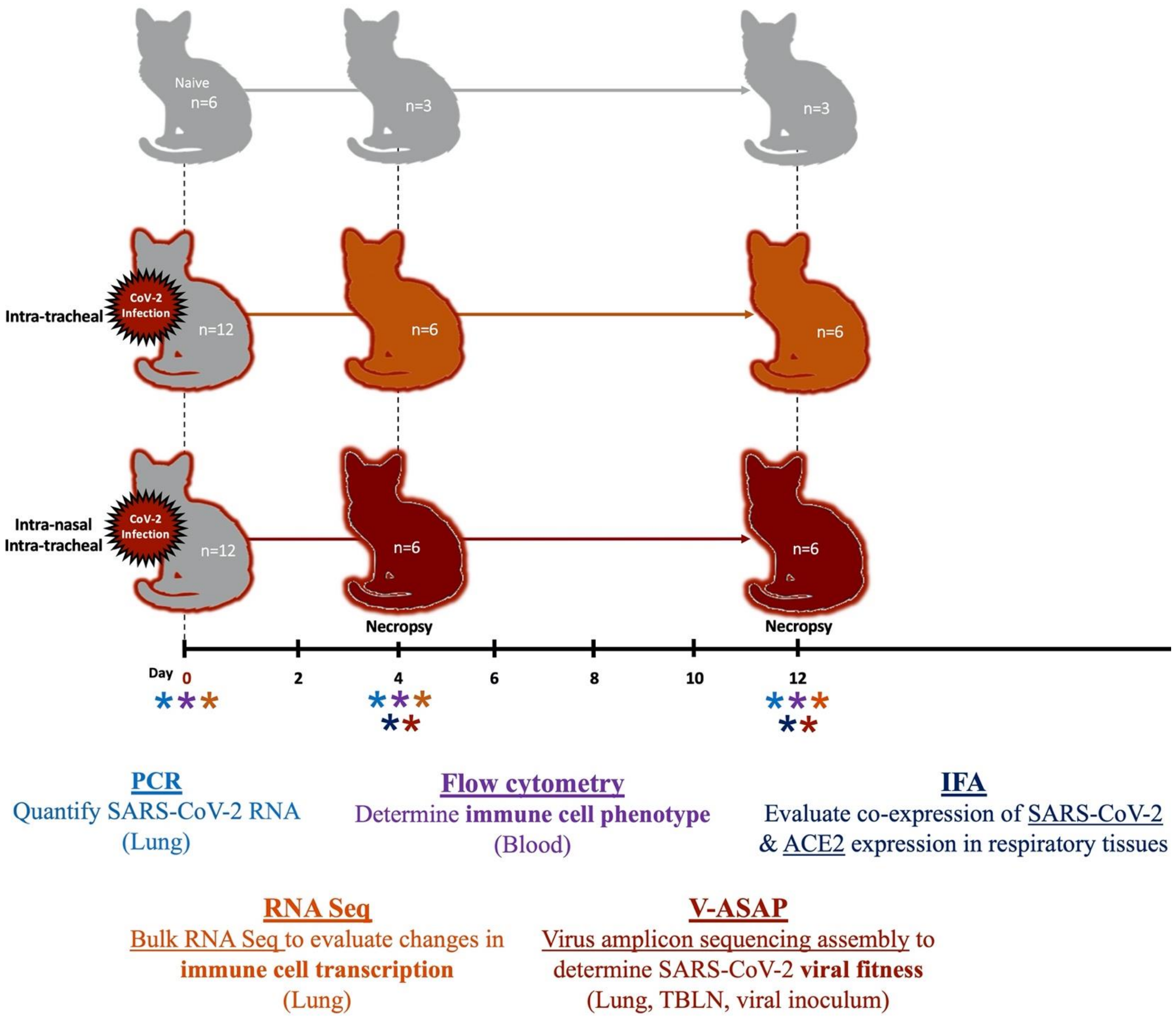

Figure S1. **Timeline of *in vivo* studies**

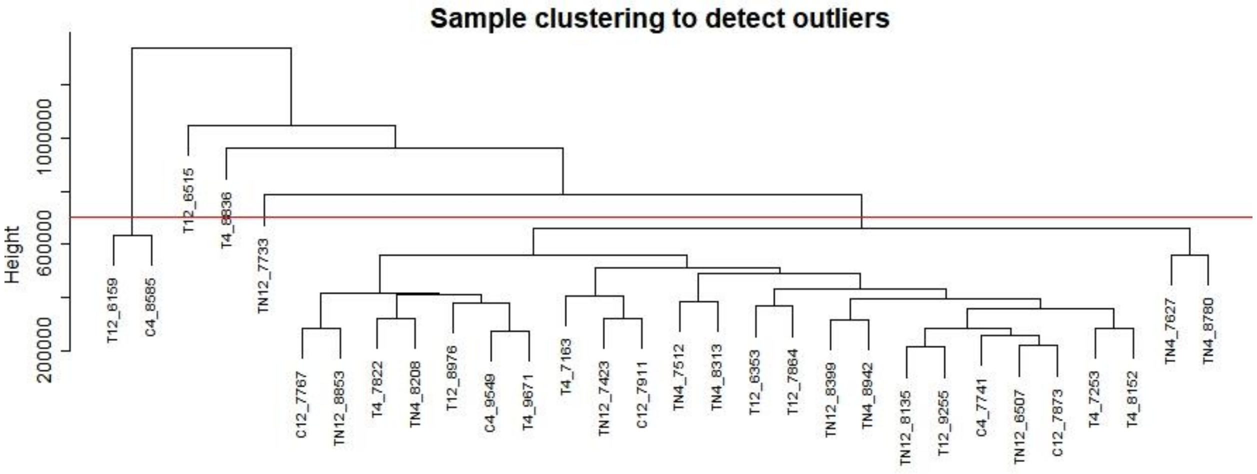

Figure S2. Clustering dendrogram of 30 samples based on their Euclidean distance. Samples are filtered for downstream analysis with a height set of 700,000.

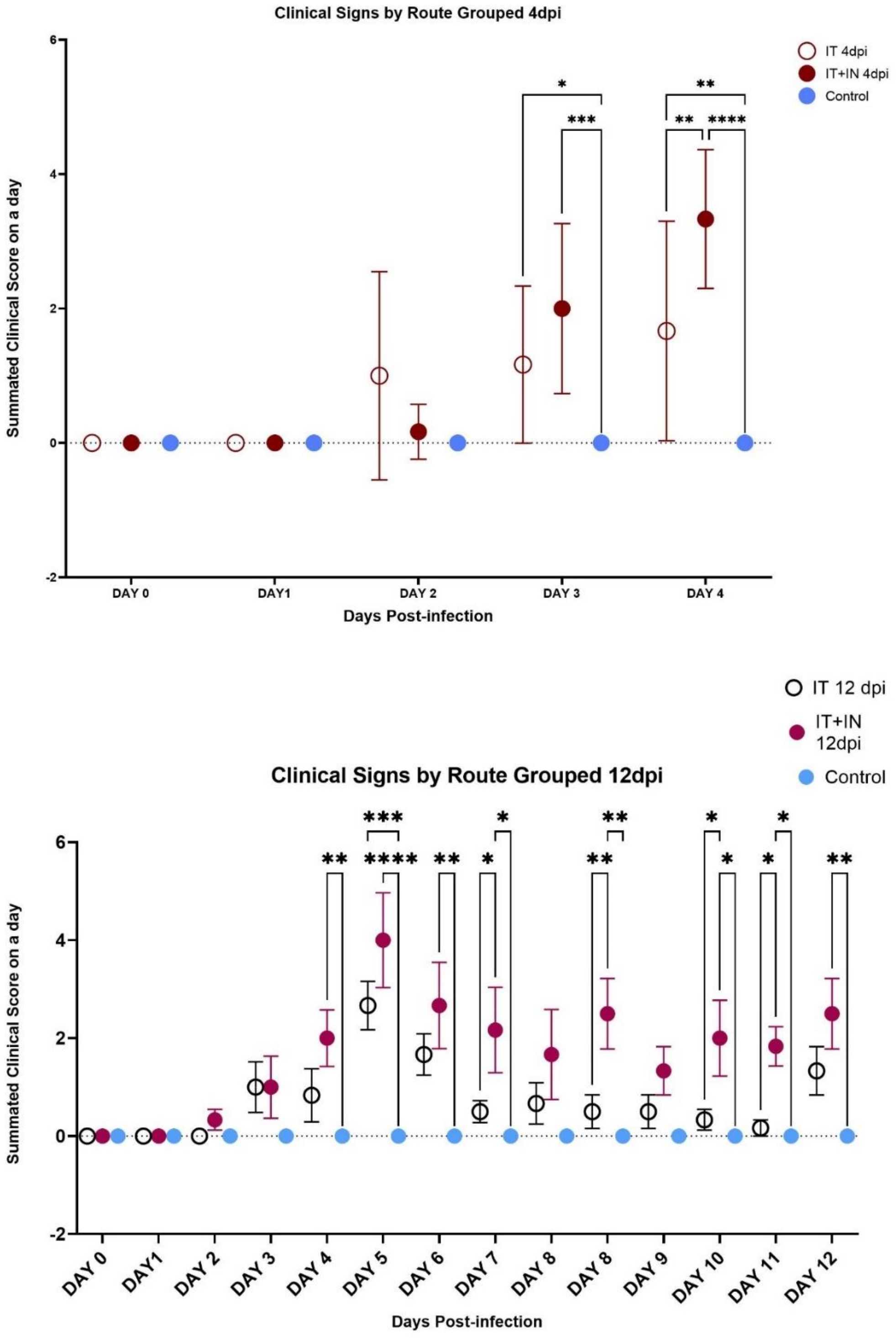

Figure S3. Clinical signs of SARS-CoV-2 cats by inoculation route. The x-axis indicates the days post-infection of the SARS CoV-2 Delta variant in cats. The y-axis shows clinical signs summated for a particular day from all the infected cats **(A)** Clinical signs recorded for both the IT route and IT+IN route in Phase1 (4dpi) show a significant increase in clinical signs in IT+IN inoculated cats compared to cats inoculated by the IT Only route on day 4 (p = 0.0013). **(B)** Clinical signs observed in Phase2 (12 dpi) are significantly increased in the IT+IN- inoculated cats compared to cats inoculated by the IT Only group on days 6, 10, and 11 (p= 0.0263). *p<0.05, **p <0.01, ***p< 0.001, ****p< 0.0001

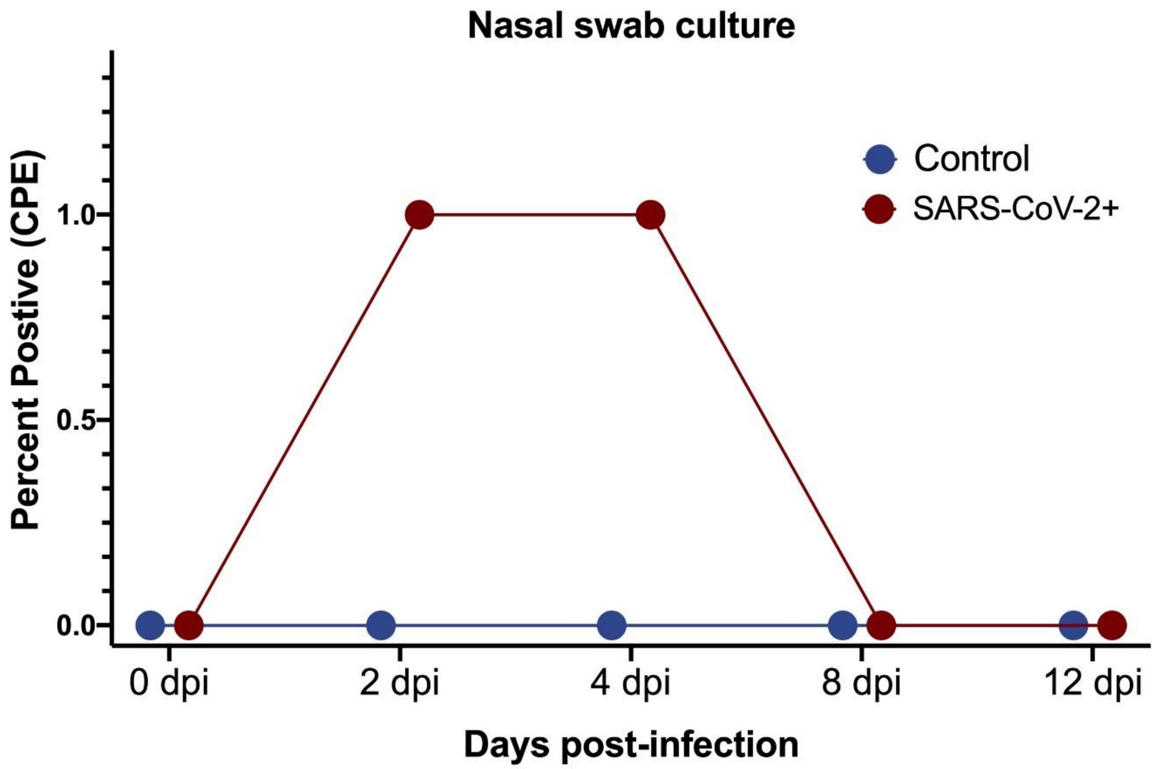

Figure S4. Virus isolation from nasal swabs during SARS-CoV-2 infection. Infectious virus was recovered from all SARS-CoV-2 (Delta) infected cats in both Phase 1 and Phase 2 of this study. Cytopathic effects were detected in cell cultures inoculated with nasal swab samples collected at 2 and 4 dpi (n=24) as compared to CPE in positive control wells.

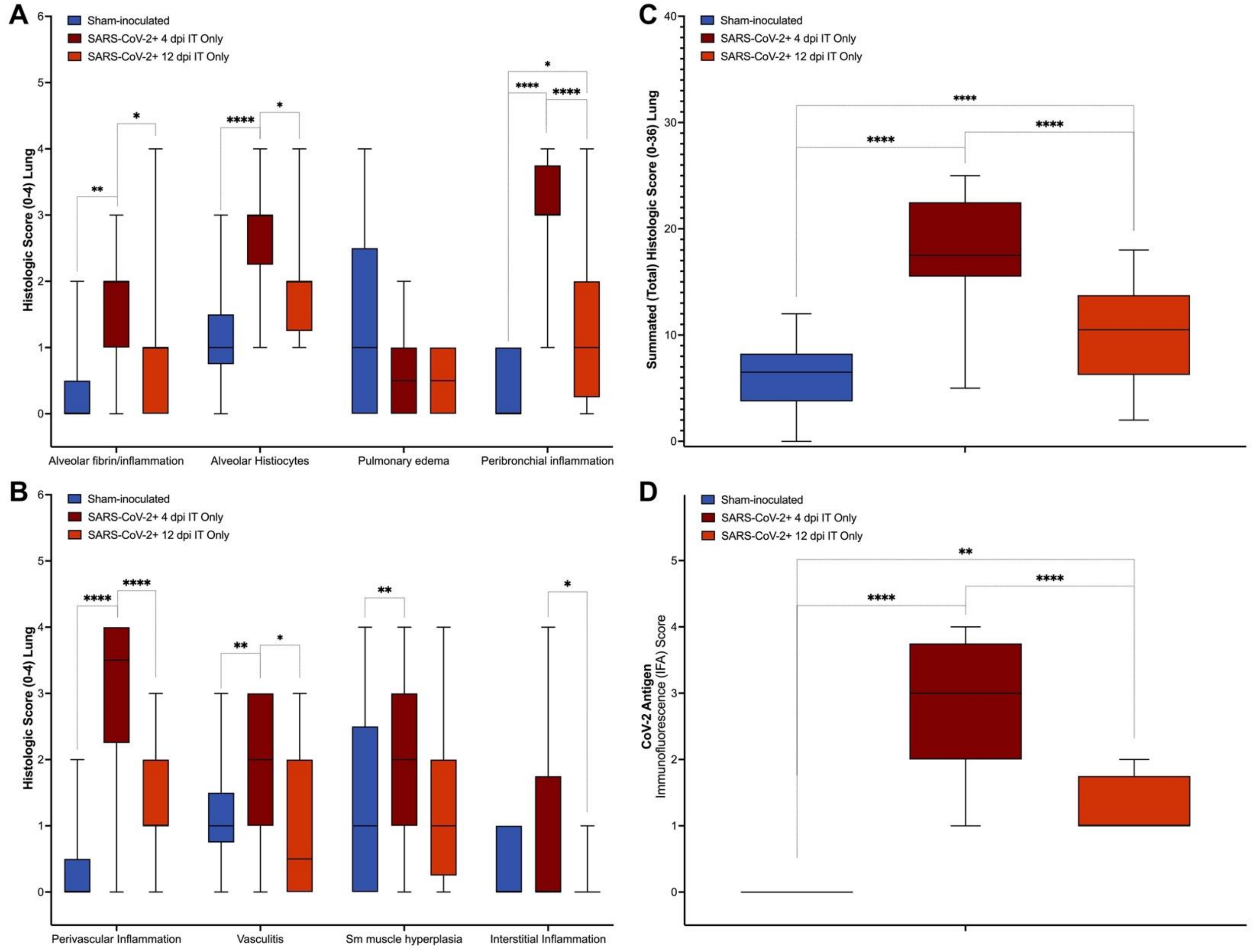

Figure S5. Histologic and immunofluorescent scoring of lung tissues in SARS-CoV-2 infected animals. (A-B) When looking at individual pathologic features, alveolar inflammation (*p*<0.05), alveolar histiocytosis *p*<0.01), and peribronchial inflammation (*p*<0.0001) were significantly increased in cats at 4 dpi compared to uninfected cats, as well as perivascular inflammation (*p*<0.0001) and vasculitis (*p*<0.05). Perivascular inflammation (p<0.001) and peribronchial inflammation (*p*<0.001) were also significantly elevated in SARS-CoV-2 infected cats at 4 dpi compared to 12 dpi. **(C)** Total lung pathology was more severe in delta-infected cats at 4 dpi compared to 12 dpi (*p*<0.0001) and uninfected controls (*p*<0.0001). Quantification of IFA fluorescent intensity revealed that the presence of viral antigen was significantly increased in CoV-2 infected cats at 4dpi when compared to uninfected controls (*p*<0.0001) and CoV-2 infected cats at 12 dpi (*p*<0.01). *p<0.05, **p < 0.01, ***p<0.001, ****p < 0.0001

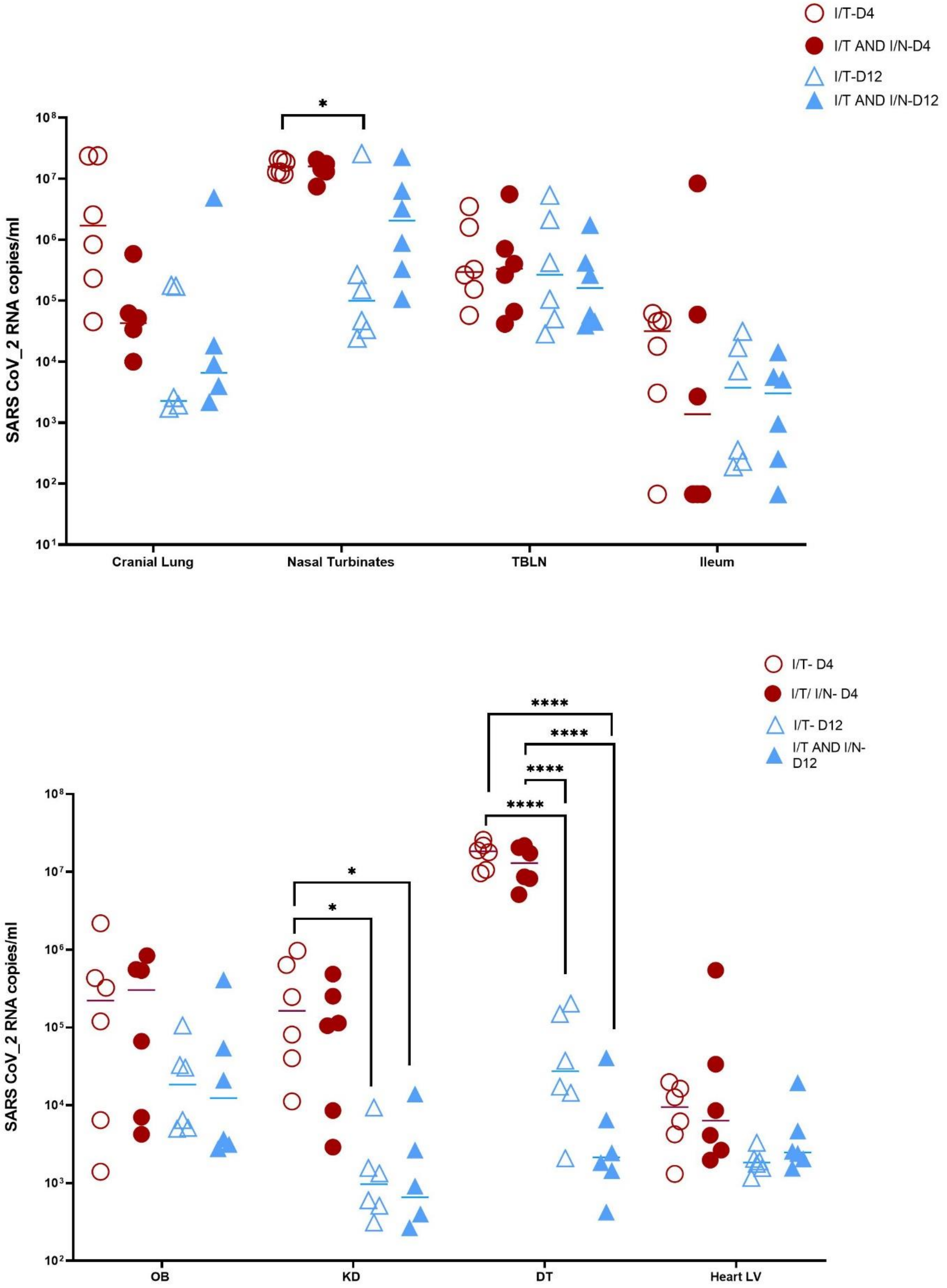

Figure S6. SARS-CoV-2 viral loads in necropsy tissues by inoculation route. Viral loads are significantly increased in the kidney (*p*=0.0478) and nasal turbinates (*p*=0.0379) of IT Only cats at 4 dpi compared to 12 dpi. In the distal trachea, there is a significant increase in viral load between 4 dpi and 12 dpi in both IN+IN ($p = < 0.0001$) and IT Only ($p = < 0.0001$) inoculated cats. No significant differences were observed between CoV-2-infected cats at either timepoint (4 dpi or 12 dpi) when evaluated by inoculation route (IN+IT vs IT Only). *p<0.05, **$p < 0.01$, ***p<0.001, ****$p < 0.0001$

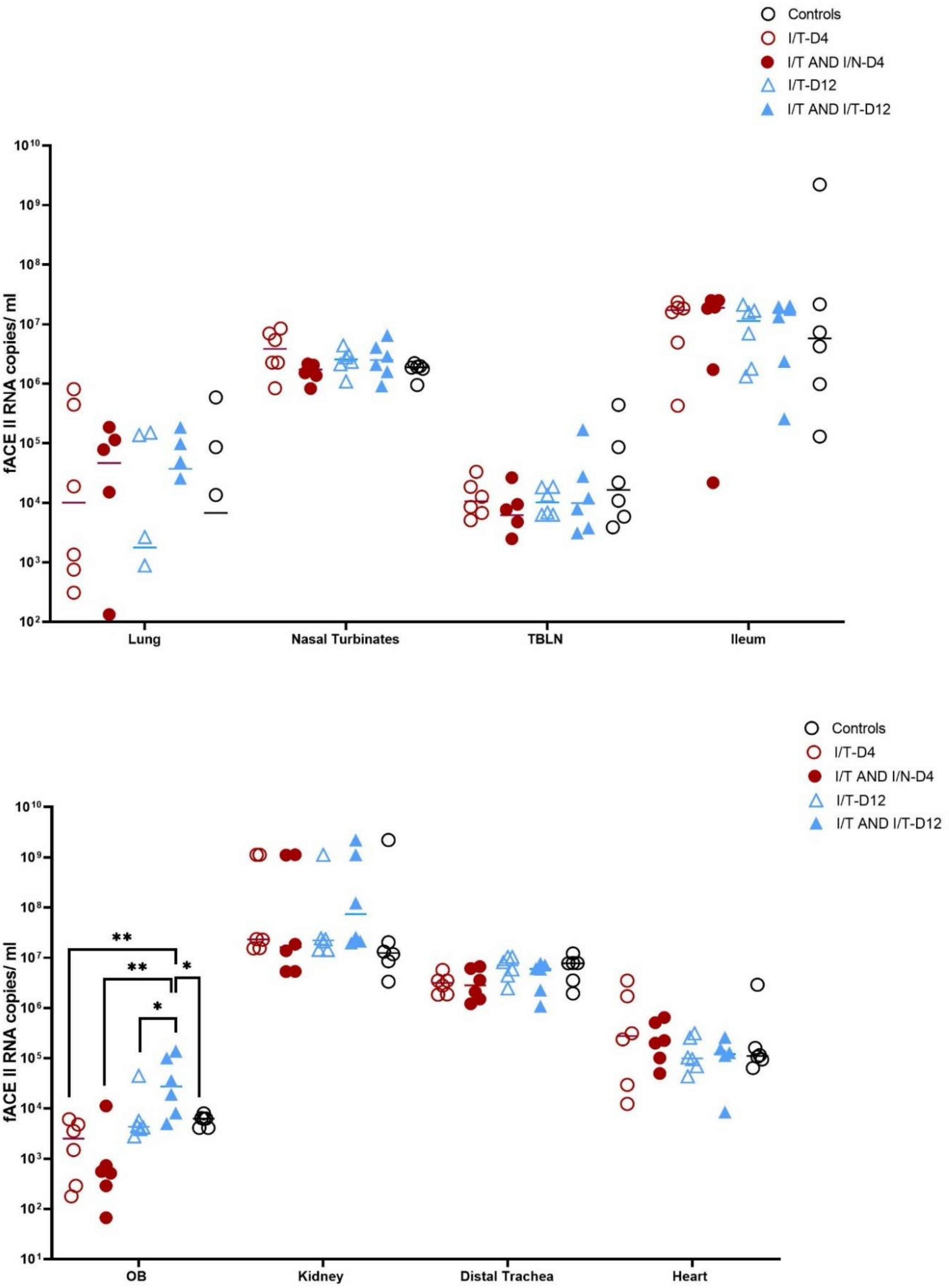

Figure S7. Feline ACE2 expression in necropsy tissues by inoculation route. f-ACE 2 expression categorized by the route of infection in each tissue showed increased f-ACE2 expression in olfactory bulb in IT+IN inoculated cats on 12 dpi compared to IT+IN inoculated cats at 4 dpi (p=0.0097). Increased f-ACE2 expression was also observed in IT Only inoculated cats at 4 dpi compared to IT+IN inoculated cats at 12 dpi (p= 0.0102), as well as IT+IN inoculated cats at 12 dpi. *p<0.05, **p < 0.01, ***p<0.001, ****p < 0.0001

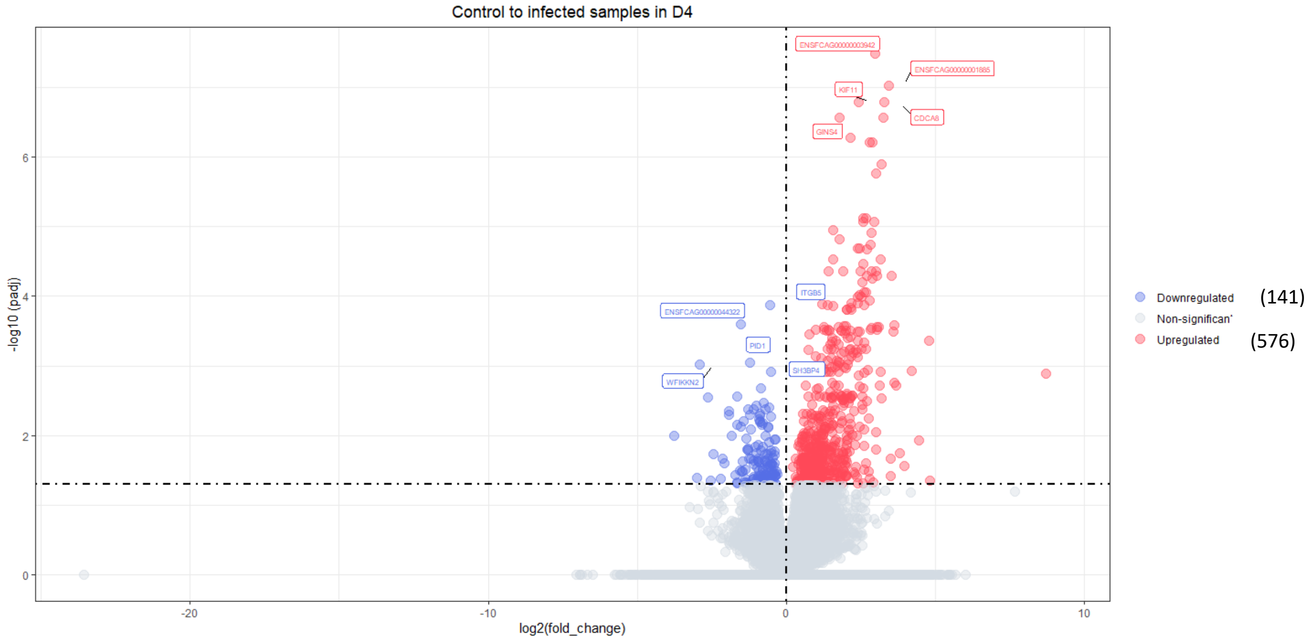

Figure S8A. Differentially expressed genes in control animals compared to CoV-2+ samples at 4 dpi. The volcano plot showing comparison of differentially expressed genes in control and Phase 1 infected samples. The genes upregulated at 4 dpi compared to controls are shown in red, whereas the genes downregulated on 4 dpi are highlighted in blue .

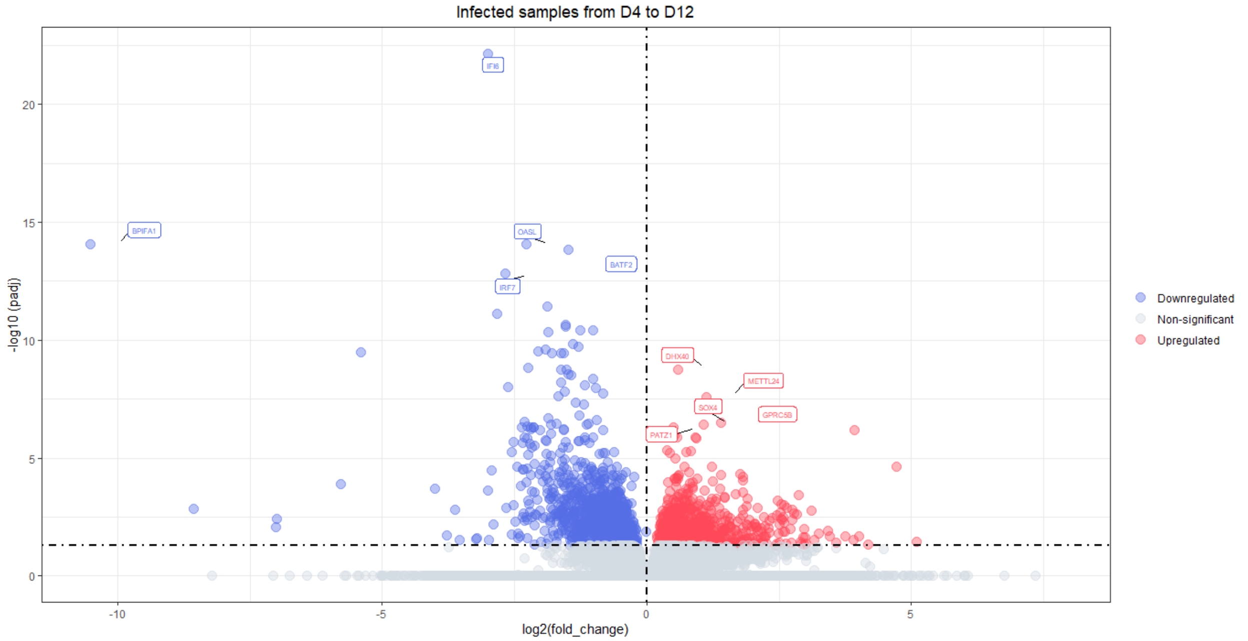

Figure S8B. Figure S8B. Differentially expressed genes in CoV-2+ samples at 4 dpi compared to 12 dpi. The volcano plot showing comparison of differentially expressed genes in Phase 1 and Phase 2 infected samples. The genes upregulated at 12 dpi compared to 4 dpi are shown in red, whereas the genes downregulated on 12 dpi are highlighted in blue .

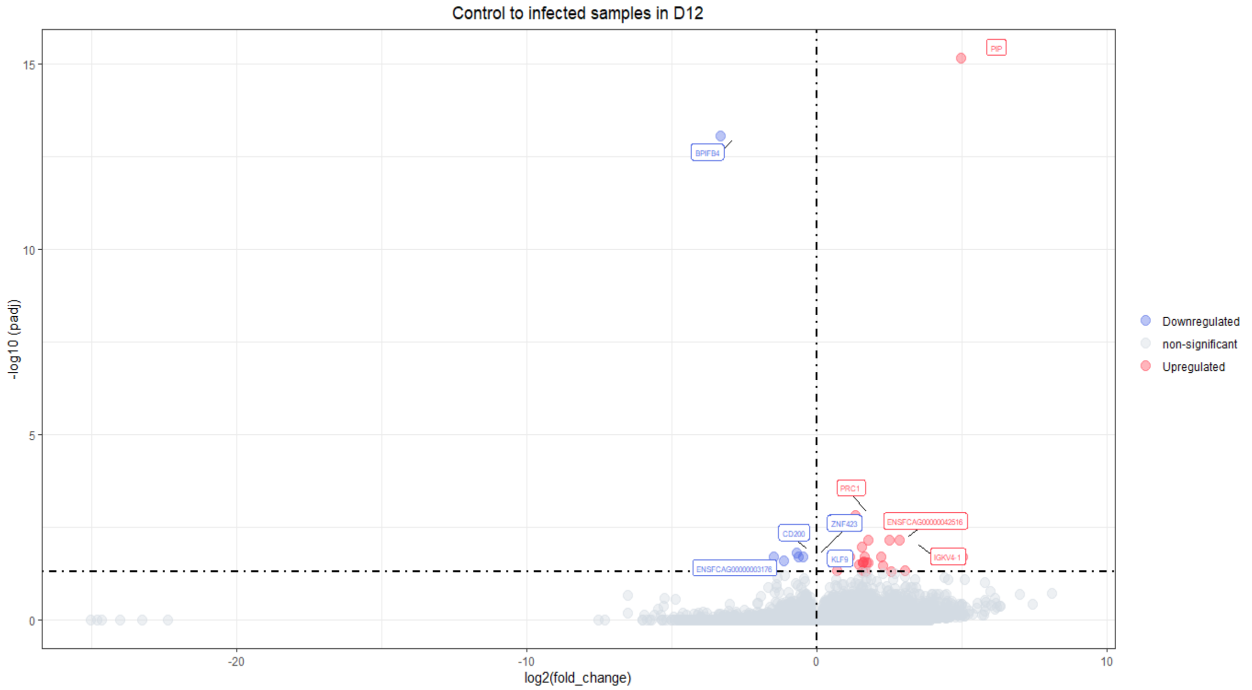

Figure S8C. **Differentially expressed genes in control animals compared to CoV-2+ samples at 12 dpi**. The volcano plot showing comparison of differentially expressed genes in control and Phase 2 infected samples. The genes upregulated at 12 dpi compared to controls are shown in red, whereas the genes downregulated on 12 dpi are highlighted in blue .

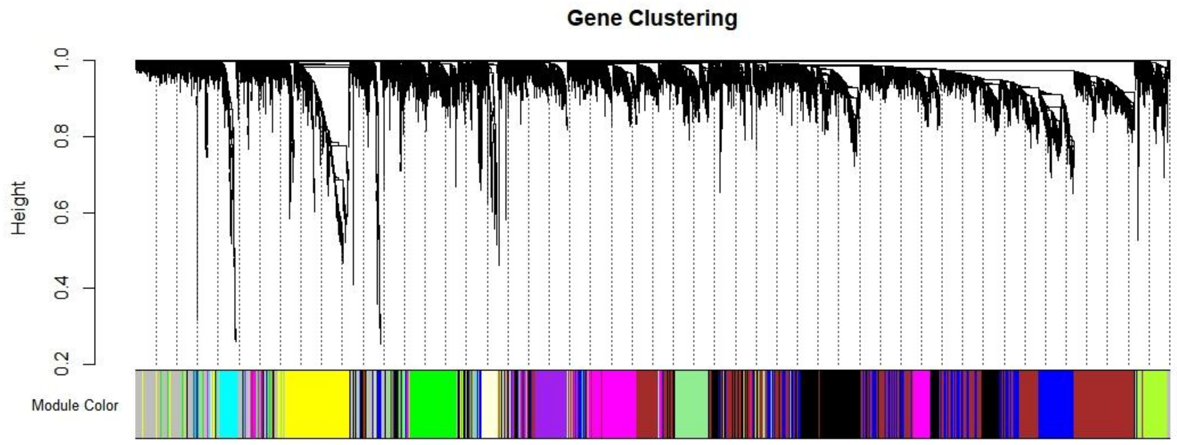

Fig 9. Gene modules detection by clustering based on the consensus expressional trend across phase 1 and 2 studies.

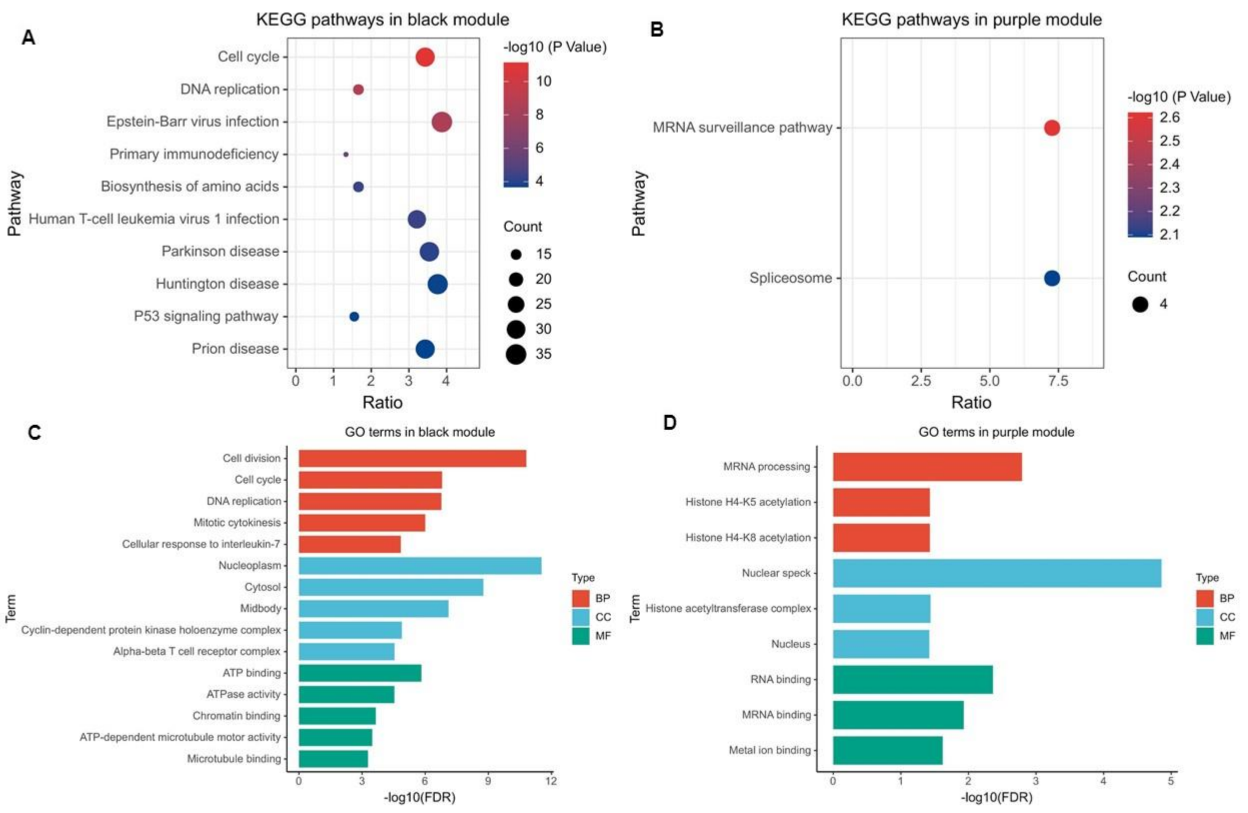

Fig S10. KEGG pathway and gene ontology term enrichment based on genes in black (A and C) and purple (B and D) modules. (A-B) KEGG Pathway analysis with DEG's of black and purple module - x-axis explains the ratio of the number of differentially expressed genes in a specific pathway compared to the total genes in a particular pathway. The y-axis is the name of the pathway involved. (C-D) GO enrichment analysis results. The x-axis shows the adjusted p-values (FDR) algorithm in each term and the y-axis represents the GO name.
